# Trend analysis of the pharmaceutical market in Iran; 1997–2010; policy implications for developing countries

**DOI:** 10.1186/2008-2231-21-52

**Published:** 2013-06-28

**Authors:** Abbas Kebriaeezadeh, Nasser Nassiri Koopaei, Akbar Abdollahiasl, Shekoufeh Nikfar, Nafiseh Mohamadi

**Affiliations:** 1 Department of Pharmacoeconomics and Pharmaceutical Administration, Faculty of Pharmacy, Tehran University of Medical Sciences, P.O. Box 14155–6451, Tehran 14174, Iran; 2 Department of Toxicology and Pharmacology, Faculty of Pharmacy, Tehran University of Medical Sciences, P.O. Box 14155–6451, Tehran 14174, Iran; 3 Management Information System Division, Osvah Pharmaceutical Company, Tehran, Iran

**Keywords:** Pharmaceutical market trends, Therapeutic categories, Pharmaceutical biotechnology, Commercialization

## Abstract

**Background:**

So far, no detailed study of the Iranian pharmaceutical market has been conducted, and only a few studies have analyzed medicine consumption and expenditure in Iran. Pharmaceutical market trend analysis remains one of the most useful instruments to evaluate the pharmaceutical systems efficiency. An increase in imports of medicines, and a simultaneous decrease in domestic production prompted us to investigate the pharmaceutical expenditure structure. On the other hand, analyzing statistics provides a suitable method to assess the outcomes of national pharmaceutical policies and regulations.

**Methods:**

This is a descriptive and cross-sectional study which investigates the Iranian pharmaceutical market over a 13-year period (1997–2010). This study used the Iranian pharmaceutical statistical datasheet published by the Iranian Ministry of Health. Systematic searches of the relevant Persian and English research literature were made. In addition, official government documents were analyzed as sources of both data and detailed statements of policy.

**Results:**

Analysis of the Iranian pharmaceutical market in the 13-year period shows that medicine consumption sales value growth has been 28.38% annually. Determination of domestic production and import reveals that 9.3% and 42.3% annual growth, respectively, have been experienced.

**Conclusions:**

The Iranian pharmaceutical market has undergone great growth in comparison with developing countries and the pharmerging group, and the market is expanding quickly while a major share goes to biotechnology drugs, which implies the need to commercialization activities in novel fields like pharmaceutical biotechnology. This market expansion has been in favor of imported medicine in sales terms, caused by the reinforcement of suspicious policies of policy makers that necessitates fundamental rearrangements.

## Background

Total expenditure on health in Iran is increasing, while the public sector’s share is decreasing. Private sector expenditure as out-of-pocket payment is remarkably high as it accounts for more than 50% of the whole expenditure [1]. The modern Iranian pharmaceutical system commenced 100 years ago with the opening of the first modern-style pharmacy by German, French, and Austrian pharmacists in Tehran. Pharmacy training was initiated by European instructors at Darolfonoon, which was remodeled with the inauguration of the Tehran Faculty of Pharmacy in 1934 which undertook a very important role in the Iranian pharmaceutical industry. Established in 1946, Abidi was the first Iranian pharmaceutical company, followed by Tolid Darou and Darou Pakhsh in 1958 and 1963, respectively [2]. After the Islamic revolution, two major motions caused fundamental changes: nationalization of the pharmaceutical industries, and generic scheme. Governmental industry privatization and transition to the semi-governmental sector was one of the major actions taken by the government in the 1988 to 1993 period. This study examines the present situation of the pharmaceutical system in Iran, including the domestic production companies, importing companies, distribution, regulations, human resources in the pharmaceutical system, and market trend analysis [3].

### Present situation of the Iranian pharmaceutical industry

Pharmaceutical production consisted mainly of 89 companies in 2010. The market concentration ratio is low. In 2010, the Herfindahl–Hirschman Index (HHI) was 295.11 for the domestic production companies, indicating little market power. Raw and packaged materials are provided by the domestic and import suppliers. Regarding sales, ten top pharmaceutical companies in 2009–2010 are shown in Table 1. Tamin Pharmaceutical Investment Company (Social Security Organization), Sobhan Pharma Group, and Shafadarou corporation (Melli Bank Investment Corporation) are the most important public owners of the pharmaceutical industry. Based on these structures, pharmaceutical holdings (Groups) sales are shown in Table 2. Data reveals an increasing rate of privatization in pharmaceutical sector.

**Table 1:** Ten top domestic production pharmaceutical companies in Iran; 2009-2010

**Rank**	**Company name**	**Market share**	**Cumulative market share**
1	Darou Pakhsh Pharma	6.6%	6.6%
2	Exir Pharma	6.1%	12.7%
3	Jaber Ebne Hayyan	5.8%	18.5%
4	Farabi	5.3%	23.8%
5	Tehran Chemie	5.2%	29.0%
6	Alborz Darou	3.8%	32.8%
7	Sobhan Darou	3.7%	36.5%
8	Osvah	3.6%	40.1%
9	Dana	2.9%	42.9%
10	Aboureihan	2.8%	45.7%

**Table 2:** Major holdings in domestic pharmaceutical production in Iran; 2009-2010

**Rank**	**Pharmaceutical holdings**	**Market share**	**Cumulative market share**
1	Tamin Investment Corporation	29%	29%
2	Sobhan Pharmaceutical Group	16%	45%
3	Shafadarou Corporation	10%	55%
4	Tehran Chimi Corporation (Private)	10%	65%

### Import companies

There are 93 private companies engaged in importing medicine, and 30 designated emergency medicine centers besides national medicine and medical equipment corporations. There is Red Crescent (Ministry of Health), Darou pakhsh trade development (Tamin Investment Corporation), and KBC (Sobhan Pharmaceutical Group), which are all public. In 2010, the HHI was 781, which is higher in comparison with that of the domestic production companies. Ten top importer companies in 2009–2010 are shown in Table 3.

**Table 3:** Ten top importer companies in Iran; 2009-2010

**Rank**	**Company name**	**Market share**	**Cumulative market share**
1	Cobel	17.2%	17.2%
2	Akbarieh	12.3%	29.6%
3	Behestan Darou	11.6%	41.2%
4	Shafayab Gostar	8.4%	49.6%
5	Jahan Behbood	5.7%	55.3%
6	Ahran Tejarat	5.0%	60.3%
7	Gostaresh Bazargani Daroupakhsh	4.8%	65.1%
8	Actover	3.4%	68.4%
9	KBC	2.6%	71.0%
10	Kavosh Gostar Darou	2.2%	73.2%

### Distribution

There were 35 nationwide drug distribution companies, among which the first four distribute approximately 70% of the entire market’s drugs. All major holdings have their own nationwide drug distribution companies that are ranked in the top four. HHI was 1387, which was significantly higher than that of the domestic production.

### Regulations

Passed in 1955, the medicine, drug, food, and drink affairs law act is the cornerstone for the current pharmaceutical procedures in Iran. Most of the bylaws and ordinances are designed and approved in the drug affairs department [2].

### Licensing

Production, importation, and distribution of medicinal products are performed under strict control by the authorities, and involve registration and licensure by the food and drug organization (formerly, Undersecretary of Food and Drug).

### Pricing

Medicine price is controlled by the food and drug organization through pricing commission regulations in a cost plus basis through comparison with selected companies according to published regulations.

### Reimbursement

Medicine cost reimbursement is mainly undertaken by three major organizations: Social Security Organization (public), Medical Services Insurance Organization (governmental), and Medical Insurance Services Organization of Armed Forces (governmental), all of which reimburse the cheapest medicine registered. The emerging supplementary insurance companies are putting constraints on pharmaceutical expenditure. There is a positive list of medicines fully covered by the government that contains specialty drugs.

### Human resources

Pharmacists are considered a major source for the pharmaceutical system. Most of the 13000 pharmacist society members are involved in pharmacies. About 6% of the pharmacists work in the industry. In the industry itself, less than 2% of the trained and scientific workforce is engaged in research and development.

### Active pharmaceutical ingredients

A growing number of companies are engaged in the production of Active Pharmaceutical Ingredient (API). Each holding has its own API-producing branch, and there are more than 30 companies engaged in production of APIs. Formal policies of the Ministry of Health actively promote motions towards independence in the API industry. Companies active in the field of API production are mainly possessed by the private sector, contrary to the finished products and distribution companies [4].

## Methods

This is a descriptive and cross-sectional study which investigates the Iranian pharmaceutical market over a 13-year period (1997–2010). This study used the Iranian pharmaceutical statistical datasheet published by the Iranian Ministry of Health. Drug consumption statistics are collected by the Food and Drug Organization (FDO) through the data received from the drug distribution companies. All distribution companies deliver their data on drug sales to the pharmacies as a unified format to the food and drug organization on a monthly basis. That data, after revision, is published as the Iranian pharmaceutical statistical datasheet yearly as sales volume (by unit) for any medicine delivered, and cost per medicine unit. Although that data does not represent real drug consumption and the subsidy paid by the government and only reveals the sales of drug to the pharmacies, it is the most unique and convenient method to monitor the pharmaceutical market. Systematic searches of the relevant Persian and English research literature were made, using electronic databases in addition to written reports. Official government documents were also analyzed as sources of data and detailed statements of policy. Different sources, such as statistics published by the Iranian Ministry of Health, pharmaceutical production companies syndicate, and the national medicine assemblies were also researched for statistics and data on medicine consumption in a domestic production and importation separation basis, production and importation statistics, pharmaceutical expenditure, medicine consumption per capita, and drug utilization by sales value in different therapeutic categories.

## Results

**Figure 1:**
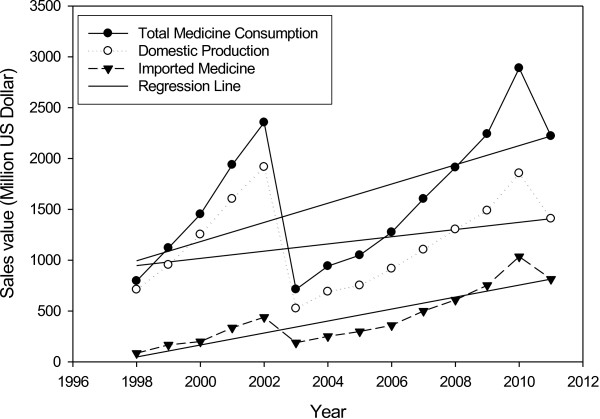
Total, domestic production and imported medicine consumption by value; 1998–2011.

**Figure 2:**
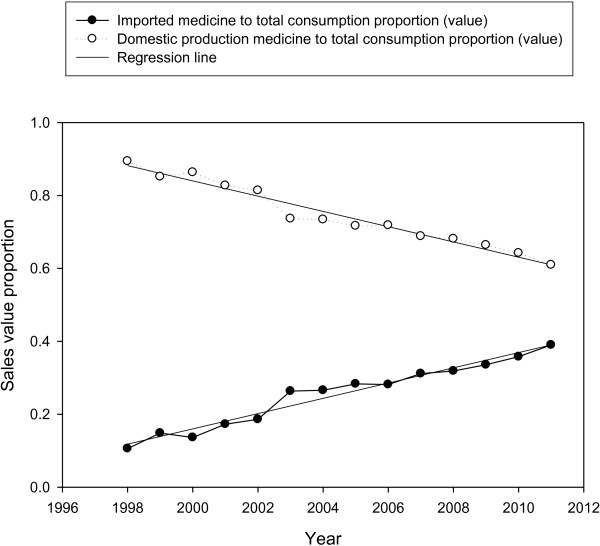
Sales value percentile proportion of imports and domestic production to the total medicine consumption; 1998–2011.

The trend analysis shows that Iranian pharmaceutical medicine market has been drastically grown in the recent decade. In the period, the population of the country has grown from 61 to 74 million, namely a total and annual growth of 21.3% and 1.53%, respectively. Total medicine market sales value reached $2.467 billion in 2010 from $0.139 billion in 1997, representing a 1669% increase (Figure 1). The annual growth rate was 28.38%. Domestic pharmaceutical production sales value reached $1.639 billion in 2010 in comparison with $0.125 billion in 1997, a 1213% growth. The average annual sales value growth is 9.3%. The correlation coefficient (R^2^) of 0.9293 shows a fairly consistent growth, though the 2011 estimate shows a decrease in market sales value. The imported medicine market sales value rose to $0.828 billion in 2010 from $0.015 in 1997, showing a dramatic total growth of 5499%, and an annual growth of 42.3%. In the period under investigation, sales value percentile proportion of imports to total drug consumption has reached 39% from 10.59% in the base year, showing a huge growth of 28.41%. The average sales value percentile proportion of imports to total medicine consumption has been 2.1% (R^2^: 0.9612) (Figure 2). The sales value percentile proportion of domestic production to total medicine consumption has reduced from 89.41% to 61% in 2010, revealing a significant decrease of 28%.

### Per capita drug consumption sales value

**Figure 3:**
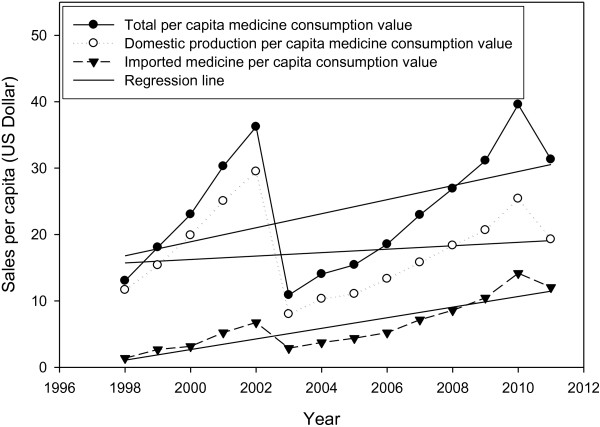
Total, domestic production and import value per capita medicine consumption; 1998–2011

Over the 13-year period, the data indicates that the index has reached $34.43 from $2.28 in 1997, which shows an overwhelming growth of 1405%, and an annual average growth of 10.8% (R^2^: 0.9235) (Figure 3). These figures show marked growth in comparison to population growth (20%).

### Per capita value of domestic pharmaceutical medicine consumption

This index has reached $22.86 from $2.05 in 1997, with a total and an annual growth of 1018% and 10.8%, respectively (R^2^: 0.9397).

### Per capita value of the imported medicine consumption

This index has reached $11.57 from $0.24 in 1997, with a total growth of 4373% (R^2^: 0.8907).

### Average sales value of the medicines by unit

**Figure 4:**
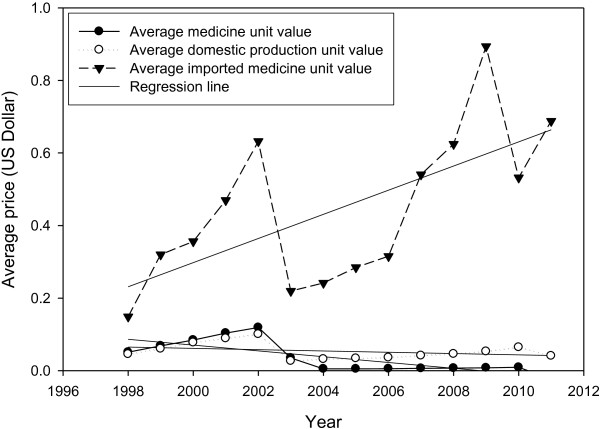
Average sales value of the total, domestic production and imported medicines by unit; 1998–2011.

The index reached an estimated $0.084 in 2011 from $0.009 in 1997, with a growth rate of 847% (R^2^: 0.9407) (Figure 4). The average sales value of domestic and imported medicines by unit also shows a total growth rate of 631% (R^2^: 0.9506) and 1930% (R^2^: 0.8345), respectively.

### Drug consumption by therapeutic categorization (by ATC codes)

**Figure 5:**
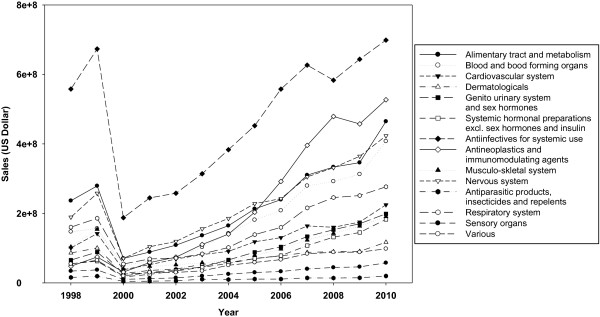
Medicine consumption by value in therapeutic categories; 1998–2010.

As the graph depicts, the market shows a point drop in the year 2000 that was due to the nationwide currency exchange rate variation (Figure 5). The data shows that antiinfectives preparations for systemic use and then antineoplastics and immunomudulating agents seize the most shares in the market and have had the steepest slope among the categories. Alimentary tract and metabolism and nervous system products also show high growth rates. The two recently mentioned categories contain the most prevalent utilization among all in terms of volume (data not shown).

## Discussion

This study presents the market environment of the pharmaceutical sector in Iran, and highlights its challenges and future prospects. In order to study a country^’^s medicine market systematically, various indicators can be applied, e.g. market behavior about specific dosage forms or quantity or quality trends [3]. Most owners of the pharmaceutical industry were either dependent on the former regime or foreigners who left Iran after the revolution, so young domestic pharmacists began to manage the pharmaceutical system of the country. Iran-Iraq War (1980–1988) also prompted the need for strict monitoring of the market. The revolutionary government exerted fundamental changes in the food and drug organization in order to assess the need for pharmaceuticals in both general society and for the military forces. With the establishment of the planning office in the food and drug organization, some experts were ascribed to closely monitor the needs and prepare the production and the import scheme. In 1986, the government in office assumed private ownership and liberalization in the economic system, which resulted in magnificent growth in the market. In 1999, the government allowed domestic production companies usage of their free capacity to cross the borders of planning by the food and drug organization. In 2001, further liberalization in the pharmaceutical system and the lack of subsidized currency caused an increase in prices. This liberalization in the pharmaceutical system also augmented imports. Throughout the period, domestic production constituted a major share in the market [3]. The major reasons for the growing demand are as follows [4,5]:

●  Iran’s demographic structure and population growth;

●  increased insurance coverage of the population;

●  increased level of income and gross domestic product (GDP) per capita;

●  incidence of new diseases and epidemiologic transition, e.g. types of cancer and multiple sclerosis;

●  intentionally lowered medicine price; and

●  medical sciences advances.

In the period under investigation, accessibility to health services and insurance coverage has grown remarkably, possibly as a result of GDP growth. The government has approached the medicine accessibility positively, and a number of medicines have been included in the medicine list. However, insurance coverage has not expanded simultaneously and proportionately. Health sector budget share in the GDP did not develop acceptably, causing a drastic increase in the public’s out-of-pocket payment. Insurance coverage expansion was through three major organizations: the Social Security Organization, Medical Services Insurance Organization, and Medical Insurance Services Organization of Armed Forces. The universal insurance coverage act obliges the population to register for an insurance service which contributes to the goal. Iranian pharmaceutical industries mostly manufacture traditional medicines, and deficiency in the investments needed for monoclonal antibodies and other biotechnology-derived products, as a consequence, helped the higher share of imported medicine. The Iranian pharmaceutical industry’s private sector has not yet developed as much as required. More than 70% of the domestic industries are owned by the government and its related bodies or the public organizations. As evidenced by the two existing biotechnology companies, both of which are privately-owned, privatization can lead to greater sustainable development in domestic industries [6,7]. Currently, total pharmaceutical market value is about $3.2 billion, or as much as $4-4.5 billion according to some estimates on the real market size [8]. The present difference between real and estimated value is attributed to the price suppression policies of the authorities [9]. It should also be noted that national currency has experienced a drastic devaluation against USD. Iran experiences an average growth rate of 28.38%, which is significantly more than developing countries such as China and India which have around 11 to 15% growth [10,11]. The results show that the domestic production sales value has grown, but not in concordance with that of the imported medicine sales value. The results also imply that the current pharmaceutical policies are in favor of imports, as the slope of the import and domestic production lines illustrate. The rise in Iranian pharmaceutical expenditure is expected to continue at a significant, even higher rate than expected from developing countries, based on factors mentioned above and the deliberate deficiencies of pharmaceutical policy [12,13]. Medicines are regarded as both public and industry goods. Iranian patients are entitled to have access to high-quality, timely, and cost-effective medicine [14,15]. The Iranian pharmaceutical market is predicted to maintain its growth for years to come. Currently, 20% of Iran’s total health expenditure, on average, goes on medicine (reimbursed by the insurance organizations, based on their reports to the supreme insurance council for last year). Antibiotics, antineoplastics and immunomudulating agents (medicines mostly used for organ transplantation rejection prohibition), antidiabetics, and alimentary tract medications gain the most value in the market (Figure 5). Regarding sales volume, analgesics, antibiotics, and second generation antidiabetics are the best sellers. Investigating the mostly used products among the two top categories it is obvious that pharmaceutical biotechnology derived medicines such as proteins and monoclonal antibodies take major positions (results not presented). Considering the items mentioned, it is concluded that new and high tech medicines are seizing a large share of the market; which should incite policy makers to change for the better. If the market continues its growth, a great financial load will be imposed on the health system. Iran contains nearly 1% of the world’s population, but only about 0.3% of the world market, so if the two are in an agreement, Iran should assume $7-8 billion in 2010, and $10-12 billion in 2020. Generic medicine use is an important component of the government’s health plan that was obligatory through the generic scheme shortly after the revolution. The policy assisted the country pass the constraints of the 1980s, but vitally restricted research and development in the domestic industry, while exerting severe price containment policies [16]. Current issues of interest for the Iranian pharmaceutical market include a rise in imports and simultaneous fall in domestic production. Low rates of investment, capital asset substitution, scarce attention to modern technologies like pharmaceutical biotechnology, and a branded medicine market are possible reasons for this domestic medicine market shrinkage [17,18]. The investment activity of pharmaceutical companies is dependant mainly on product demand, profits, technological developments, and capital availability. It is vital to notice the steady decrease in investment rate for capital equipment replacement. This is associated with the market shift towards imported medicines and the lack of well-designed, long-term policies to support the domestic pharmaceutical industry that would potentiate reasonable conditions to encourage domestic production [19]. Meanwhile vast research projects have been conducted in the universities and research centers but few of them have been commercialized into medicines. Performing studies aligned with the needs of the biopharmaceutical industry could be seen as a choice. Policy makers and authorities could contribute the national production capabilities by establishing the basements to commercialize the researches, revising the policies, forming close relationships and cooperative committees with the biopharmaceutical industries and applying evidence based decision making procedures.

## Conclusions

The absence of significant basic and applied biopharmaceutical research that can result in industrial breakthroughs and attain competitive advantage in Iran has induced a state of regression in the domestic industry that has accelerated a drain on the capital and energy of the industry. Such conditions, along with price suppression, increase imports [20,21]. It is advised that policy makers take all necessary measures towards establishing an appropriate pharmaceutical business environment that favors domestic production, as well as controlling rising expenditure in the pharmaceutical sector [22]. We can conclude that the average value of imported medicine per unit is growing, which suggests technological content is in continuous improvement in comparison with domestic ones [23-25]. Medicine consumption per capita shows that this index is high in Iran. Therefore, it is proposed that conducting applied research projects ordered by the biopharmaceutical industries along with preparing the requirements for commercialization of novel fields like pharmaceutical biotechnology which assigns a great share in the national pharmaceutical expenditure be in the top agenda.

## Abbreviations

HHI: Herfindahl–Hirschman Index; API: Active Pharmaceutical Ingredient; FDO: Food and Drug organization; GDP: Gross Domestic Product; ATC: Anatomical Therapeutic Chemical Classification System.

## Competing interests

The authors declare that they have no competing interests.

## Authors’ contributions

AK conceived the strategy of study and supervised the project, NNK conceived and implemented the strategy, performed the data analysis and statistical interpretation and drafted the paper, AA gave consultation on designing the study, complemented the data and statistical analysis, SN gave consultation on the study methodology and edited the draft, NM performed the data analysis. All authors read and approved the final manuscript.
